# An Echocardiographic Evaluation of Dilated Cardiomyopathy in a Tertiary Care Hospital

**DOI:** 10.31729/jnma.3992

**Published:** 2019-02-28

**Authors:** Raj Kumar Thapa, K.C. Kanchan, Rishi Khatri, Devendra Khatri, Rajeeb K. Deo, Drishti Shah

**Affiliations:** 1 Department of Medicine, Shree Birendra Hospital, Chhauni, Kathmandu, Nepal; 2 Department of Emergency and General Practice, Shree Birendra Hospital, Chhauni, Kathmandu, Nepal

**Keywords:** *dilated cardiomyopathy*, *echocardiography*, *ejection fraction*, *left ventricle*

## Abstract

**Introduction:**

Cardiomyopathies are diseases of heart muscle that may originate from genetic defects, cardiac myocyte injury or infiltration of myocardial tissues. Dilated cardiomyopathy is the most common phenotype and is often a final common pathway of numerous cardiac insults. Mostly it remains unknown in the absence of echocardiography, histopathology and genetic evaluation. Though common it is underdiagnosed with not much of data available in our setup.

**Methods:**

This study was analytical cross-sectional study of hospital data on Echocardiographic findings in 65 patients of DCM visiting cardiology unit for Echocardiographic evaluation from 1^st^ of February to 31^st^ July 2018 for the period of six months in Shree Birendra Hospital, a tertiary care military hospital at Chhauni, Kathmandu. Pediatric age group patients and those who refused to give consent were excluded. Data obtained were entered in Microsoft Excel 2010 and analyzed by IBM SPSS 21.

**Results:**

Among 65 patients enrolled 40 (61%) were male and 25 (39%) female with male to female ratio of 1.6:1. Elderly people (61-75 years) with an average age of 65 were commonly involved and they presented mostly with congestive heart failure, 32 (49%). Echocardiographic evaluation showed 36 (55%) with mildly dilated Left Ventricle (5.6-6.0cm). Majority had reduced Left ventricular systolic function with an average Ejection fraction (EF) of 39.6%. No significant difference between male and female with the average EF% (P=0.990) and there was no significant relation between age and average EF% (P=0.091).

**Conclusions:**

Dilated Cardiomyopathy is the commonest cardiomyopathy phenotype mostly presenting with congestive heart failure. It is often underdiagnosed in our part of the world, however echocardiography will easily detect the condition.

## INTRODUCTION

Cardiomyopathies (CM) are diseases of heart muscle. Various etiologies such as genetic defects, cardiac myocyte injury and infiltration of myocardial tissues leads to CM. Dilated cardiomyopathy (DCM) is the most common type with enlargement of one or both of the ventricles resulting in systolic dysfunction with reduced ejection fraction (EF%).^1–5^ The natural history of DCM is incompletely understood as it is caused by various agents resulting in variable presentations.^1,6^

The annual mortality rate has a wide range between 10% and 50%.^7^ DCM approximately accounts for 25% of the Congestive heart failure (CHF) cases and remaining 75% are due to ischemic, hypertensive or non-systolic heart failure.^8,9^ Genetic and specific studies in highly specialized centers can identify causes in about 50% cases and remaining are the diagnosis of exclusion, termed idiopathic variants.^10–12^

The aim of this study is to determine incidence, and evaluate Echocardiographic findings in patients of DCM.

## METHODS

An analytical cross-sectional study was done utilizing hospital data on Echocardiographic findings in 65 patients of DCM visiting cardiology unit for Echocardiographic evaluation from 1^st^ of February to 31^st^July 2018 for the period of six months in Shree Birendra Hospital (SBH), a tertiary care military hospital at Chhauni, Kathmandu. Diagnosis of DCM was done on the basis of history, clinical features (symptoms & signs) and Echocardiographic evaluation. Data from register book were retrieved after ethical approval from institutional review committee. Standardized questionnaire developed by the researcher was used to obtain clinical and socio-demographic characteristics of the patients after informed consent. Pediatric age group patients and those who refused to participate were excluded.

Maximum internal diameter of left ventricle (LV) during diastole less than or equal to 5.5cm considered normal. M-mode measurement of LV during systole and diastole used for calculating Ejection fraction (EF) where EF% less than 50% considered reduced. Left ventricular diastolic dysfunction (LVDD) grade one defined by E/A ratio less than one and grade two as E/A ratio more than one (pseudo normal) with some structural LV filling defect (where, E = peak velocity blood flow from gravity in early diastole and A = peak velocity flow in late diastole caused by atrial contraction).

Echocardiography was performed by Cardiologists to see cardiac structural and functional abnormalities. Machine used was SIEMENS Model: ACUSON SC2000 having the facility of two dimensional view, M-mode and color flow Doppler imaging. Data obtained were entered in Microsoft Excel 2010 and analyzed by software IBM SPSS 21. Statistical significance between male and female with EF was calculated by means t-test whereas relationship between age and EF by ANOVA (analysis of variance). P<0.05 was considered significant.

## RESULTS

**Figure 1.**
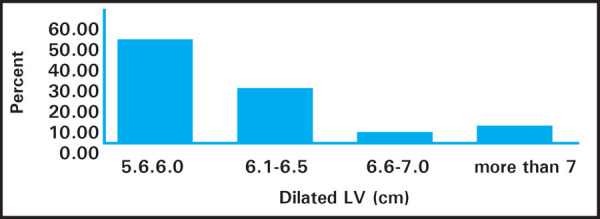
Left ventricular dilatation in echocardiography.

Among 65 patients enrolled 40 (61%) were male and 25 (39%) female with male to female ratio of 1.6:1. Most of the cases have unknown etiology 53 (81%). On Echocardiography, 36 (55%) had mildly dilated Left Ventricle (5.6-6.0cm) (Figure 1). Additionally, dilated Left Atrium was found in 15 (23%) cases and dilated Right Atrium along with Right Ventricle in 12 (18%).

**Figure 2.**
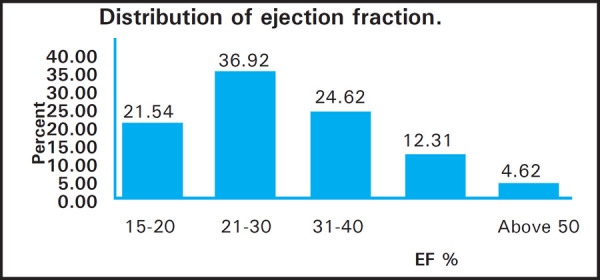
Distribution of ejection fraction.

Functional Mitral regurgitation in 48 (74%) with Grade I Left ventricular diastolic dysfunction in 56 (86%). Majority had reduced Left ventricular systolic function with Ejection fraction between 21% and 30%, an average of 39.6% (Figure 2).

**Figure 3.**
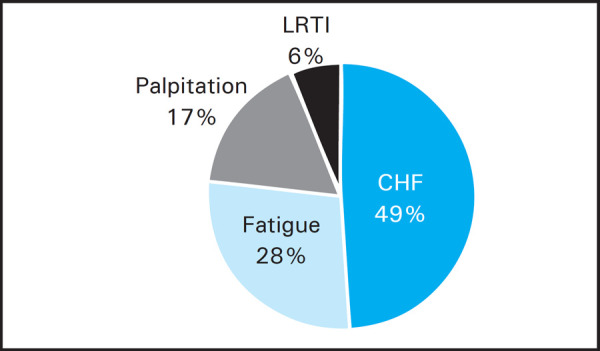
Common clinical presentations.

Elderly age group (61-75 years) with an average age of 65, were encountered frequently 34 (52%) and the most common clinical presentation was congestive heart failure 32 (49%) (Figure 3).

Besides, mild pericardial effusion found in 2 (3%) and none of our cases had any intra-cavitary thrombus or vegetation.

On applying two sample means ‘t-test’ there was no statistical significant difference between average EF% of both male and female (P = 0.990) (Table 1).

**Table 1.** Descriptive statistics of ejection fraction scores.

Gender	N	Mean	Std. Deviation	Std. Error Mean	P
Male	40	39.6250	14.90945	2.35739	0.990
Female	25	39.5833	9.19790	1.87751	

Similarly, by ANOVA (Analysis of Variance) there was no relationship of age and average EF% (P = 0.091) (Table 2).

**Table 2.** Analysis of variance.

	df	SS	MS	F	Significance F
Regression	1	481.0212539	481.0213	2.942835	0.091253213
Residual	62	10134.21312	163.4551		
Total	63	10615.23438			

## DISCUSSION

DCM was more common in male than female (61% vs 39%) and frequent on the elderly with the mean age of 65 years in our study population. In the study done by Suha MA et al.^13^ 53% were male and 47% female, similar to our study with males outnumbering females. Most of our patients presented with congestive heart failure (49%) supported by Animasahun BA et al.^14^ where it was 54.5%. Similar result was evident in the study by Towbin JA et al.^12^

Dilated Left ventricle (LV) is mandatory for the diagnosis of DCM, however Right ventricle (RV) may similarly be affected, Mathew T et al.^2^ In our study association of RV dilation was 18%.

Incidence of functional Mitral Regurgitation (MR) in our study was 48%. Functional MR, which occurs as a consequence of regional or global LV dysfunction despite a structurally normal mitral valve, is a common complication in patients with DCM, Jun K et al,^3^ Meese RB et al,^15^ Ballester M et al,^16^ Chandraratna PA et al.^17^ and its presence predicts poor outcome, Donal E et al.^18^

Some degree of Left ventricular diastolic dysfunction (LVDD) is inevitable in DCM ranging from grade one to four. We had 86% cases with LVDD grade one. Assessment of diastolic function in DCM also correlated by studies Nishimura RA et al,^19^ Lavine SJ et al,^20^ Appleton CP et al.^21^ and Pinamonti B et al.^22^

LV systolic dysfunction quantified by measuring Ejection fraction (EF) in percentage and the estimated EF% assumes uniform or global left ventricular function, Shah PM.^23^ Also evidenced byFolland ED et al.^24^ and Stamm RB et al.^25^

None of our cases had intra-cavitary thrombus, however it is a common complication in patients with DCM, Asinger RW et al.^26^ and Gottdiener JS et al.^27^ The limitations of our study being the data of a single center and lack of etiologic evidence.

## CONCLUSIONS

Patients commonly present with symptoms and signs of congestive heart failure and a simple Echocardiography will easily detect the condition. However, etiologic diagnosis is not possible mostly in a resource limited settings. More research works to be done to understand this common heart failure condition and our aim should be to diagnose preclinical DCM cases and thereby reduce significant mortality and morbidity.
